# Clinical Characteristics and Outcomes of Colorectal Cancer in the ColoCare Study: Differences by Age of Onset

**DOI:** 10.3390/cancers13153817

**Published:** 2021-07-29

**Authors:** Caroline Himbert, Jane C. Figueiredo, David Shibata, Jennifer Ose, Tengda Lin, Lyen C. Huang, Anita R. Peoples, Courtney L. Scaife, Bartley Pickron, Laura Lambert, Jessica N. Cohan, Mary Bronner, Seth Felder, Julian Sanchez, Sophie Dessureault, Domenico Coppola, David M. Hoffman, Yosef F. Nasseri, Robert W. Decker, Karen Zaghiyan, Zuri A. Murrell, Andrew Hendifar, Jun Gong, Eiman Firoozmand, Alexandra Gangi, Beth A. Moore, Kyle G. Cologne, Maryliza S. El-Masry, Nathan Hinkle, Justin Monroe, Matthew Mutch, Cory Bernadt, Deyali Chatterjee, Mika Sinanan, Stacey A. Cohen, Ulrike Wallin, William M. Grady, Paul D. Lampe, Deepti Reddi, Mukta Krane, Alessandro Fichera, Ravi Moonka, Esther Herpel, Peter Schirmacher, Matthias Kloor, Magnus von Knebel-Doeberitz, Johanna Nattenmueller, Hans-Ulrich Kauczor, Eric Swanson, Jolanta Jedrzkiewicz, Stephanie L. Schmit, Biljana Gigic, Alexis B. Ulrich, Adetunji T. Toriola, Erin M. Siegel, Christopher I. Li, Cornelia M. Ulrich, Sheetal Hardikar

**Affiliations:** 1 Huntsman Cancer Institute, Salt Lake City, UT 84112, USA; caroline.himbert@hci.utah.edu (C.H.); jennifer.ose@hci.utah.edu (J.O.); tengda.lin@hci.utah.edu (T.L.); lyen.huang@hsc.utah.edu (L.C.H.); Anita.peoples@hci.utah.edu (A.R.P.); courtney.scaife@hci.utah.edu (C.L.S.); Bartley.Pickron@hsc.utah.edu (B.P.); Laura.Lambert@hci.utah.edu (L.L.); jessica.cohan@hsc.utah.edu (J.N.C.); mary.bronner@aruplab.com (M.B.); Swanson.Eric@scrippshealth.org (E.S.); Jolanta.Jedrzkiewicz@hsc.utah.edu (J.J.); Neli.ulrich@hci.utah.edu (C.M.U.); 2 Department of Population Health Sciences, University of Utah, Salt Lake City, UT 84112, USA; 3 Cedars-Sinai Center, Los Angeles, CA 90048, USA; jane.figueiredo@cshs.org (J.C.F.); hoffmand@toweroncology.com (D.M.H.); Yosef.Nasseri@cshs.org (Y.F.N.); deckerr@toweroncology.com (R.W.D.); Karen.zaghiyan@cshs.org (K.Z.); Zuri.murrell@cshs.org (Z.A.M.); andrew.hendifar@cshs.org (A.H.); jun.gong@cshs.org (J.G.); efiroozmand@yahoo.com (E.F.); alexandra.gangi@cshs.org (A.G.); Beth.Moore@cshs.org (B.A.M.); kyle.cologne@med.usc.edu (K.G.C.); maryliza.el-masry@csmns.org (M.S.E.-M.); 4 Department of Surgery, University of Tennessee Health Science Center, Memphis, TN 37996, USA; dshibata@uthsc.edu (D.S.); natehinkle27@gmail.com (N.H.); jmonroe1@uthsc.edu (J.M.); 5 H. Lee Moffitt Cancer Center and Research Institute, Tampa, FL 33612, USA; seth.felder@moffitt.org (S.F.); Julian.sanchez@moffitt.org (J.S.); sophie.dessureault@moffitt.org (S.D.); domenico.coppola@fdhs.com (D.C.); Stephanie.Schmit@moffitt.org (S.L.S.); Erin.Siegel@moffitt.org (E.M.S.); 6 Department of Surgery, Washington University St. Louis, St. Louis, MO 63130, USA; mutchm@wustl.edu (M.M.); cbernadt@wustl.edu (C.B.); deyali@wustl.edu (D.C.); a.toriola@wustl.edu (A.T.T.); 7 Fred Hutchinson Cancer Research Center, Seattle, WA 98109, USA; mssurg@uw.edu (M.S.); shiovitz@uw.edu (S.A.C.); ulrik.wallin@polyclinic.com (U.W.); wgrady@fredhutch.org (W.M.G.); plampe@fredhutch.org (P.D.L.); dreddi@uw.edu (D.R.); mkrane@uw.edu (M.K.); Ravi.Moonka@vmmc.org (R.M.); cili@fredhutch.org (C.I.L.); 8 Department of Laboratory Medicine and Pathology, University of Washington, Seattle, WA 98195, USA; 9 Baylor Scott & White Health, Dallas, TX 76712, USA; Alessandro.Fichera@BSWHealth.org; 10 Pathologisches Institut, University Hospital Heidelberg, 69120 Heidelberg, Germany; Esther.Herpel@med.uni-heidelberg.de (E.H.); Peter.Schirmacher@med.uni-heidelberg.de (P.S.); matthias.kloor@med.uni-heidelberg.de (M.K.); magnus.knebel-doeberitz@med.uni-heidelberg.de (M.v.K.-D.); johanna.nattenmueller@med.uni-heidelberg.de (J.N.); hans-ulrich.kauczor@med.uni-heidelberg.de (H.-U.K.); Biljana.Gigic@med.uni-heidelberg.de (B.G.); aulrich@lukasneuss.de (A.B.U.)

**Keywords:** early onset, colorectal cancer, cohort, epidemiology

## Abstract

**Simple Summary:**

The number of new colorectal cancer cases continues to increase in individuals under 50 years of age in the Western world. Underlying reasons for this observation remain unclear. Here, we compare demographic, clinical, and lifestyle characteristics by age at diagnosis in a large cohort of newly diagnosed colorectal cancer patients. We aim to identify potential risk factors and clinical characteristics of colorectal cancer patients diagnosed under the age of 50 years, compared to those over 50. The results of this study will help elucidate factors related to colorectal cancer in younger patients, and may help guide future research on colorectal cancer in younger patients.

**Abstract:**

Early-onset colorectal cancer has been on the rise in Western populations. Here, we compare patient characteristics between those with early- (<50 years) vs. late-onset (≥50 years) disease in a large multinational cohort of colorectal cancer patients (*n* = 2193). We calculated descriptive statistics and assessed associations of clinicodemographic factors with age of onset using mutually-adjusted logistic regression models. Patients were on average 60 years old, with BMI of 29 kg/m^2^, 52% colon cancers, 21% early-onset, and presented with stage II or III (60%) disease. Early-onset patients presented with more advanced disease (stages III–IV: 63% vs. 51%, respectively), and received more neo and adjuvant treatment compared to late-onset patients, after controlling for stage (odds ratio (OR) (95% confidence interval (CI)) = 2.30 (1.82–3.83) and 2.00 (1.43–2.81), respectively). Early-onset rectal cancer patients across all stages more commonly received neoadjuvant treatment, even when not indicated as the standard of care, e.g., during stage I disease. The odds of early-onset disease were higher among never smokers and lower among overweight patients (1.55 (1.21–1.98) and 0.56 (0.41–0.76), respectively). Patients with early-onset colorectal cancer were more likely to be diagnosed with advanced stage disease, to have received systemic treatments regardless of stage at diagnosis, and were less likely to be ever smokers or overweight.

## 1. Introduction

An emerging concern in colorectal cancer is the rapidly rising incidence among those under the age of 50 years (early-onset patients) [1,2,3]. Since 1990, early-onset colorectal cancer has significantly increased globally and the number of new cases is expected to increase by 140% by the end of 2030 [4,5]. In response, the American Cancer Society and the United States Preventive Services Task Force (USPSTF) guidelines have been recently updated to advocate for initiating colorectal cancer screening at the age of 45 years [6,7]. Drivers of the recent increase in early-onset colorectal cancer have yet to be identified, although established modifiable risk factors for late-onset colorectal cancer including diet, obesity, low physical activity, and smoking are potential key players [8,9,10,11].

Accumulating evidence suggests that distinct biological characteristics and mechanisms underlie the development of early-onset colorectal cancer as compared to colorectal cancer diagnosed among individuals over 50 years old [12]. Genetic profiles of patients with early-onset colorectal cancer still remain unclear [12]. About 30% of early-onset cases can be attributed to family history and hereditary conditions, although these are not hypothesized to drive the increasing incidence in this population [12]. Early-onset colorectal cancers are more likely to be microsatellite stable, and investigators continue to discover chromosomal abnormalities specific to early-onset disease [12]. Recently, our team has discovered deregulated redox homeostasis as one molecular phenotype of early-onset colorectal cancer patients [13]. Dysbiosis of the gut microbiome is another hypothesized molecular driver of the early-onset colorectal cancer burden [12]. External (e.g., stress, antibiotics, diet, etc.) and internal (e.g., inflammation) elements throughout life can alter gut microbiome health and may affect the risk of developing colorectal cancer in early years [12]. Key pathways within these hypothesized biological mechanisms that are associated with early-onset disease have yet to be discovered [12].

To date, clinical recommendations for colorectal cancer treatment do not differ by age of onset [14]. Yet, prior studies have observed that a more aggressive treatment regimen is generally adopted for early-onset patients as compared to late-onset patients [15,16,17,18]; in particular, an increased use of systemic treatments, including neoadjuvant and adjuvant therapy, are reported. Regardless of tumor stage and treatment regimens, survival among the early-onset patient population seems to be superior to the older population [19]. Hence, whether or not early-onset patients experience a greater benefit and less side effects from these systemic treatments remains unclear [15,16,17,18].

Here, we describe demographic (age, sex, race, ethnicity), clinical (stage at diagnosis, site, treatment), and lifestyle (smoking status, body mass index (BMI)) characteristics of a large international cohort of prospectively followed patients with colorectal cancer, with the goal of identifying potential risk factors and clinical correlates of early-onset colorectal cancer.

## 2. Materials and Methods

### 2.1. Study Population

The design and population of the ColoCare Study (www.clinicaltrials.gov (accessed on 19 July 2021), Identifier: NCT02328677) have previously been described [20,21,22,23,24]. Briefly, the ColoCare Study is a multicenter international prospective cohort recruiting patients with newly diagnosed colorectal cancer at any stage (International Classification of Diseases, 10th edition, C18–C20). Patients are recruited at multiple sites in the United States [Fred Hutchinson Cancer Research Center (FHCRC, Seattle, WA, USA); H. Lee Moffitt Cancer Center and Research Institute (Moffitt, Tampa, FL, USA); University of Tennessee Health Science Center (UTHSC, Memphis, TN, USA); Washington University School of Medicine (WUSM, St. Louis, MO, USA); Huntsman Cancer Institute (HCI, Salt Lake City, UT, USA); Cedars-Sinai Medical Center (Cedars, Los Angeles, CA, USA)] and in Germany (University of Heidelberg(HBG, Heidelberg, Germany)).

In the current analysis, we report data on *n* = 2193 men and women recruited in the ColoCare Study cohort from December 2009 through to March 2020, with detailed data from questionnaires and medical chart abstractions. The study was approved by the Institutional Review Boards of the respective recruitment sites, and all patients provided written informed consent.

### 2.2. Data Collection

Questionnaires administered at study enrollment (baseline) assessed demographic (age at diagnosis, sex, race/ethnicity) and behavioral (smoking, BMI) characteristics. Clinical characteristics including stage at diagnosis and primary tumor site, recurrence, vital status, and adjuvant and neoadjuvant treatment were abstracted from medical records.

### 2.3. Data Elements

Demographic characteristics: Patients were categorized by age of onset (<50 and ≥50 years) at the time of diagnosis, ethnicity (Hispanic and non-Hispanic) and race (White, African American, and other, which includes Asians, Native Hawaiians, Native Americans, and patients reporting belonging to more than one race).

Tumor and clinical characteristics: Patients were grouped by stage at diagnosis (0, I, II, III, IV—before receipt of any neoadjuvant treatment), tumor site based on ICD-10 codes (colon = C18.0–C18.9; rectum = C19.9, C20.9, C21.8), recurrence status at 2 years after surgery (“yes” = had recurrence, “no” = no recurrence), vital status (“alive”, “deceased”), and neo (rectal cancers only) and/or adjuvant treatment (“yes” = received neo/adjuvant treatment, “no” = did not receive neo/adjuvant treatment).

Behavioral characteristics: Patients were categorized by their smoking behavior (current, former, never smoker). BMI was computed using anthropometric measurements (kg/m^2^). BMI categories were computed following the World Health Organization (WHO) categorization (underweight: ≤18.5 kg/m^2^, normal weight: >18.5 to <25 kg/m^2^, overweight: ≥25 kg/m^2^ to <30 kg/m^2^, obese: ≥30 kg/m^2^).

### 2.4. Statistical Analyses

Mean and standard deviation (SD) values were calculated for continuous variables (age, BMI). Frequencies and percentages were calculated for categorical variables (age of onset, sex, race, ethnicity, tumor stage, tumor site, recurrence, vital status, neoadjuvant and adjuvant treatment, smoking, BMI categories). Clinical characteristics were compared by age of onset (early- vs. late-onset) and tumor site. Furthermore, we also compared the clinical characteristics by tumor site within age groups. We have currently modeled all missing data as a separate category in our statistical models.

Multivariate logistic regression models (odds ratio (OR) and 95% confidence interval (95% CI)) were computed to assess the independent associations of stage at diagnosis, tumor treatment (neoadjuvant (rectal cancer patients only) and adjuvant), smoking, and BMI categories with age of onset. The primary outcome was early-onset colorectal cancer in each model. ORs and 95% CI were computed for three models: (1) adjusted for sex and race; (2) adjusted for sex, race, tumor site, and stage at diagnosis; and (3) adjusted for sex, race, tumor site, stage at diagnosis, smoking, BMI, and study site, respectively. These variables were parameterized as outlined above. Some studies have suggested a varying risk for intermediate onset colorectal cancers [25]; therefore, subgroup analyses were conducted categorizing patients into early- (<50 years), intermediate (50–55 years), and late-onset (>55 years). All statistical analyses were performed using SAS (Version 9.4, SAS, Cary, NC, USA) software.

## 3. Results

Patient characteristics, overall and by age of onset, are summarized in Table 1. Overall, 82% of patients were recruited at one of the American sites, while 18% were recruited in Germany. Fifty-seven percent were male, and the mean age was 60 years (SD ± 13 years). The majority of the cohort reported being non-Hispanic (93%) and White (87%). Of note, the initial recruitment into the ColoCare Study occurred in Germany, and the entire 18% of our German cohort was European White, impacting the overall racial/ethnic distribution. Overall, there were approximately equal numbers of colon and rectal cancers (52% vs. 47%, respectively). Study participants were diagnosed predominantly with stage II or III (60%) colorectal cancers. In a subset with detailed treatment information abstracted (75% of the cohort), 34% and 43% of the study population received neoadjuvant and/or adjuvant treatments, respectively. At the time of this analysis, 20% were deceased, and 15% had experienced a colorectal cancer recurrence. A larger proportion of patients were never smokers (40%), and overweight or obese (60%); the mean BMI of the cohort was 28.6 kg/m^2^ (±6 kg/m^2^).

Twenty-one percent (*n* = 459) of patients were diagnosed with early-onset colorectal cancer (Table 1). Early-onset colorectal cancer patients were more likely to be of Hispanic ethnicity compared to late-onset patients (8% vs. 4%), with no differences by race or sex. Early-onset patients were more likely to be diagnosed at a more advanced stage (III or IV) (early-onset: stage III: 40%, stage IV: 23%; late-onset: stage III: 34%, stage IV: 17%). They were also more likely to receive adjuvant and/or neoadjuvant treatment (neoadjuvant: 84% vs. 68%, adjuvant: 50% vs. 41% in early-onset compared to late-onset patients, respectively). A lower proportion of early-onset cancers were deceased at the time of the current analyses (14% vs. 21% early- vs. late-onset patients, respectively), while the proportion of recurrence was about 15% in both groups. Early-onset patients were less likely to be overweight (25%) or obese (28%) as compared to late-onset patients (36% and 32%, respectively), while the proportion of underweight patients was higher in early- vs. late-onset cancers (16% vs. 7%). Patients diagnosed with early-onset cancers were more likely to be never smokers (45%) in comparison with late-onset colorectal cancers (39%).

Table 2 summarizes patient characteristics comparing early- and late-onset patients by tumor site. When further categorized by tumor site, the cohort consisted of 18% early-onset and 82% late-onset colon cancers, and 21% early-onset and 79% late-onset rectal cancer patients. Forty-nine percent of early-onset patients were diagnosed with colon cancers as compared to 54% of late-onset patients, while 51% of early-onset patients were rectal cancers as compared to 46% of late-onset patients. The proportion of women was slightly higher among early-onset (51%) compared to late-onset colon cancer patients (47%). Early-onset rectal cancer patients had a slightly lower proportion of deceased patients compared to late-onset rectal cancer patients (11% vs. 20%). Among early-onset patients, the proportion of current and former smokers was higher among patients with rectal cancer compared to those with colon cancer (42% vs. 29%).

Regardless of tumor site, early-onset patients were more likely to receive neoadjuvant and/or adjuvant treatments. Table 3 compares proportions of early- vs. late-onset colon cancer patients receiving adjuvant treatment by stage at diagnosis. A higher proportion of stage II and III early-onset colon cancer patients received adjuvant treatment (45% and 90%, respectively) as compared to late-onset colon cancer patients (27% and 85%, respectively). Table 4 compares proportions of early- vs. late-onset rectal cancer patients receiving neoadjuvant and adjuvant treatment by stage at diagnosis. A slightly higher proportion of early-onset stage I rectal cancer patients received neoadjuvant treatment (40% vs. 32%). Overall, a higher proportion of early-onset rectal cancer patients tend to receive neoadjuvant treatment as compared to late-onset rectal cancer patients (stage I: 40% vs. 32%, stage II: 82% vs. 71%, stage III: 91% vs. 79%, stage IV: 85% vs. 76%), even when such treatment may not be clinically indicated, e.g., for stage I rectal cancer patients where neoadjuvant treatment is not the standard of care. Similar observations were observed for adjuvant treatment among rectal cancer patients.

Table 5 summarizes the results of adjusted logistic regression models assessing associations between patients with early-onset colorectal cancer and stage at diagnosis, tumor treatment (neoadjuvant, adjuvant), smoking, and BMI categories. All the results described below are from a model (Model 3) which is mutually adjusted for sex, race, tumor site, stage at diagnosis, BMI, smoking, and study site. The odds of being diagnosed with a more advanced stage for early-onset patients was approximately two times that of late-onset patients (stage III: 1.99 (1.39–2.87), stage IV: 2.50 (1.69–3.72)). Among rectal cancer patients, the odds of receiving neoadjuvant treatment was 2.31-fold (1.43–3.70) higher for early-onset compared to late-onset patients. Similar results were observed for adjuvant treatment. Early-onset colorectal cancer patients were less likely to be overweight and obese compared to patients with late-onset colorectal cancer (OR (95% CI) comparing early- to late-onset = 0.56 (0.41–0.76) and 0.66 (0.48–0.90), for overweight and obese patients, respectively). Early-onset patients were more likely to be never smokers compared to late-onset patients (OR (95% CI) = 1.55 (1.21–1.98)).

Some studies have suggested a varying risk for intermediate onset colorectal cancers [25]; therefore, we conducted further subgroup analyses categorizing patients into early- (<50 years), intermediate (50–55 years), and late-onset (>55 years) colorectal cancer (Supplementary Table S1). Similar to early-onset patients, the odds of receiving adjuvant treatment were 1.54-fold (1.15–2.05) for patients with intermediate onset disease as compared to late-onset disease. Intermediate onset patients were more likely to be diagnosed with stage IV disease (1.79-fold (1.16–2.78)) compared to late-onset patients. No differences were observed for stage 0–III. Similar to early-onset patients, the odds of being a never smoker were 1.54-fold (1.19–2.01) for patients with intermediate onset disease in contrast with late-onset disease. No differences between intermediate and late-onset patients were observed for neoadjuvant treatment or BMI. These results indicate that cancers developing early in life may have distinct risk factors compared to those developing later in life.

## 4. Discussion

This study describes demographic, clinical, and behavioral characteristics of participants in the ColoCare Study, an international multicenter cohort of patients with newly diagnosed colorectal cancer. Patients with early-onset colorectal cancer were observed to be diagnosed at a higher stage compared to their late-onset counterparts. Additionally, regardless of stage at diagnosis and tumor site, early-onset patients were more likely to receive a more aggressive treatment regimen than the recommended standard of care compared to late-onset patients, e.g., stage I rectal cancer patients received neoadjuvant treatment when not indicated by National Comprehensive Cancer Network (NCCN) guidelines. Early-onset patients were generally “healthier” and were less likely to be smokers or overweight/obese.

The most recently updated colorectal cancer treatment guidelines from NCCN do not include different recommended regimens for early- vs. late-onset colorectal cancers [14]. Yet, it has been observed that early-onset patients are generally subjected to more aggressive treatment regimens [15,16,17,18]. A large cohort study of 1424 patients with early-onset and 10,810 with late-onset colorectal cancer recently reported that 12% of patients with stage I colorectal cancer in their cohort received systemic treatments [15], despite the fact that the NCCN guidelines do not recommend any systemic treatment for patients with stage I colorectal cancer [14]. This observation was also reported in other large population-based studies [16,18]. One study particularly observed a higher prescription of adjuvant chemotherapy among early-onset patients at all stages without gaining any survival benefit [16]. Results from our study support these observations and indicate that (1) early-onset patients—regardless of stage at diagnosis or tumor site—are more likely to receive systemic treatment, and (2) stage I rectal cancer patients seem to receive neoadjuvant and/or adjuvant treatment regardless of age of onset. Occasionally, multidisciplinary treatment teams espouse applications for neoadjuvant chemoradiation beyond locally advanced stage II and III rectal cancers. One such indication is for earlier stage (e.g., stage I, T2N0) distal rectal cancers for which response may increase the likelihood of sphincter preservation. Younger patients are often the ideal candidates for this approach due to multifactorial reasons, including aggressive interest in avoiding permanent colostomy, ability to tolerate multimodality therapy, and strong pre-existing baseline bowel function that would translate to acceptable function/continence following aggressive sphincter-preserving surgery. Systemic treatments, however, are highly toxic and may cause severe short- and long-term complications including cumulative neuropathy and liver toxicity [26]. Understanding patterns of treatments among this high-risk subgroup will aid further evaluation and appropriate adjustment of treatment guidelines.

Modifiable risk factors for early-onset colorectal cancer have yet to be established. Given the strong evidence for the obesity–colorectal cancer relationship, studies on colorectal cancer in young individuals have early on investigated the impact of obesity on the observed increased incidence in this population [10,27]. To date, results remain limited and inconclusive. We observed a lower proportion of overweight and obese patients within our early-onset cancers compared to late-onset cancers. However, a recently published study comparing BMI of 269 patients with early-onset and 2809 with late-onset colorectal cancer did not support our findings, and reported similar BMI distributions in the early- and late-onset groups [10]. While BMI is an established risk factor for colorectal cancer overall, our data does not identify BMI as the driver for the increased incidence of early-onset colorectal cancer. Underlying reasons may be that BMI has been more strongly associated with colon cancer, and the observed association seems to be stronger for men than for women [28].

Smoking has been strongly associated with increased overall colorectal cancer risk in previous studies [29,30]. Zisman et al. observed that smokers were on average 5.2 years (95% CI: 4.9–5.5. years) younger at their colorectal cancer diagnosis than non-smokers. Two studies have investigated smoking as a driver for early-onset colorectal cancer [10,31]. While one study did not observe differences in smoking behavior, a more recent study supports our findings of early-onset patients being less likely to be smokers [10,31]. Taken together, our results suggest that the traditional risk factors for CRC such as BMI and smoking may not explain the recent increase in early-onset cancers, demonstrating the need to identify other risk factors that may explain this increasing trend of early-onset cancers.

The observed rise in the incidence of patients with early-onset colorectal cancer has been reported to be largely driven by rectal and left-sided colon cancers [4,15]. Burnett-Hartman et al. reported a higher proportion of rectal cancers in patients under the age of 39 years [15]. However, underlying reasons for such an increase remain unknown. We did not observe such an increase in rectal cancers in younger patients in our cohort, although we observed a trend for a higher incidence of colon cancer among female early-onset patients.

Our results are aligned with previous studies reporting that patients with early-onset colorectal cancer are characterized by more advanced disease stage at diagnosis [4,12,32]. Delayed diagnosis or misdiagnosis with other related colorectal diseases in the younger population, no existing recommended guidelines for targeted screening, varying symptoms, as well as other unknown molecular factors are hypothesized to underlie this observation [4,12,32,33]. Recently, societies, including the American Cancer Society and the USPSTF [6,7], updated their guidelines to lower the colorectal cancer screening initiation age to 45 years, which may help reduce the number of cases with advanced stage disease among the early-onset colorectal cancer population.

Compared to the general US population, our study population consisted of both a higher proportion of early-onset (21%) and rectal (47%) cancers, making the ColoCare Study cohort a great resource to study these high-risk subgroups. In comparison, out of the expected new patients with colorectal cancer in the US in 2020, approximately 12% will be early-onset, and 29% will be rectal cancers [34]. Our study population was largely recruited at National Comprehensive Cancer Centers and University clinics. Some of these specialized centers are more likely to treat referrals and complex surgeries, which may partly explain the higher proportion of rectal and early-onset patients in our cohort. In the US, on average, over 40% of individuals across all age groups are obese (BMI ≥ 30 kg/m^2^) [35]. Our study population had slightly lower rates of obesity for patients under and over the age of 50 years.

This study has several strengths and limitations. The ColoCare Study is an observational cohort and the reported associations may be influenced by unmeasured confounding. Our study population had a higher proportion of rectal and early-onset patients as compared to the general population, making it an ideal research environment to study these high-risk subgroups, including patients with early-onset cancer. Previous research assessing treatment differences by age of onset in patients with colorectal cancer were limited to data from the United States [9,15,33]; having an international study site is particularly unique to our cohort, and allows comparisons between treatment trends in patients with early-onset colorectal cancer. Tumor and clinical characteristics were abstracted from medical records, ensuring accurate classifications of the study population. Family history—as well as other molecular tumor features including MSI status, which have previously been associated with early-onset disease—could not be included in the present study due to pending medical chart abstractions on a larger proportion of our study participants. As smoking behavior is self-reported in the questionnaires, there may be misclassification of smoking status due to social desirability or recall bias.

## 5. Conclusions

This study of a large prospectively followed colorectal cancer cohort revealed differences in stage at diagnosis and site, neo and adjuvant treatment, BMI, and smoking behavior among patients with early-onset colorectal cancer compared to late-onset patients. Studies comparing treatment differences in patients with early- and late-onset colorectal cancer are needed to test the risk-benefit of more aggressive treatment regimens for patients with early-onset colorectal cancer. Future research involving a more comprehensive assessment of newer modalities of tobacco use, including the use of e-cigarettes, are needed to completely understand the contribution of tobacco use to the recent increase of colorectal cancer in younger patients. More accurate assessments of body composition, including the proportion of visceral and subcutaneous adipose tissues, trends over time in body composition measures, and childhood obesity and weight fluctuations should be considered over BMI when investigating the impact of obesity on early-onset colorectal cancer.

## Figures and Tables

**Table 1:** Demographic, tumor, treatment, and behavior characteristics of the ColoCare Study cohort by age of onset ^A^.

		Early-Onset Colorectal Cancer (<50 Years) (*n* = 459)		Late-Onset Colorectal Cancer (≥50 Years) (*n* = 1734)		Total (*n* = 2193)	
		*n*	21%	*n*	79%	*n*	%
Demographic Characteristics							
Sex	Male	252	55	1000	58	1293	57
	Female	207	45	734	42	941	43
Ethnicity	Hispanic	35	8	63	4	98	4
	Non-Hispanic	409	89	1649	95	2058	94
Race	White	386	84	1528	88	1914	87
	African American	28	6	114	7	142	6
	Other *	36	8	80	5	66	6
Tumor and Clinical Characteristics							
Stage at diagnosis	0	7	2	61	4	68	3
	I	50	11	322	19	372	17
	II	93	20	436	25	529	24
	III	185	40	597	34	782	36
	IV	107	23	291	17	398	18
Tumor site	Colon	209	45	933	54	1142	52
	Rectum	219	48	799	46	1018	47
Recurrence	Yes	67	14	252	15	320	15
	No	261	57	1134	65	1395	63
Vital status	Alive	392	85	1365	79	428	20
	Deceased	64	14	364	21	1757	80
Neoadjuvant Treatment (rectal only)	Yes—Total	159	84	437	68	596	72
	No	30	16	201	32	231	28
Adjuvant treatment	Yes—Total	232	50	704	41	936	43
	No	94	21	577	33	671	31
Behavioral Characteristics							
Smoking	Current	64	14	208	12	272	12
	Former	95	21	638	37	733	33
	Never	207	45	679	39	886	40
BMI, mean (SD)		28 (7)		29 (6)		29 (6)	
BMI categories	Underweight	62	13	95	5	195	9
	Normal weight	142	31	434	25	576	26
	Overweight	116	25	617	36	733	33
	Obese	128	28	561	32	689	31

* “Other” includes Asians, Native Hawaiians, Native Americans, and patients reporting belonging to more than one race. ^A^ Data has not yet been abstracted on *n* = 37 (2%) for ethnicity, *n* = 21 (1%) for race, *n* = 56 (3%) for tumor stage, *n* = 44 (2%) for tumor site, *n* = 478 (22%) for recurrence, *n* = 8 (0.3%) for vital status, *n* = 621 (27%) for receipt of neoadjuvant treatment, *n* = 585 (26%) for receipt of adjuvant treatment, *n* = 302 (14%) for smoking, and *n* = 157 (7%) for BMI.

**Table 2:** Frequencies and percentages of patient, tumor, and behavior characteristics by tumor site and age of onset ^A^.

		Colon Cancer (*n* = 1142)				Rectal Cancer (*n* = 1018)			
		Early-Onset (*n* = 209)		Late-Onset (*n* = 933)		Early-Onset (*n* = 219)		Late-Onset (*n* = 799)	
		*n*	%	*n*	%	*n*	%	*n*	%
Demographic Characteristics									
Sex	Male	103	49	434	47	132	60	500	63
	Female	106	51	499	53	87	40	299	37
Ethnicity	Hispanic	15	7	36	4	19	9	27	3
	Non-Hispanic	190	91	882	94	193	88	765	96
Race	White	167	80	804	86	189	86	722	90
	African American	18	9	80	9	10	5	34	4
	Other *	19	7	43	5	16	7	37	5
Tumor and Clinical Characteristics									
Stage at diagnosis	0	5	2	31	4	2	1	30	4
	I	27	13	191	20	18	8	130	16
	II	42	20	255	27	45	21	181	23
	III	65	31	259	28	113	52	228	42
	IV	65	31	183	20	38	17	108	14
Recurrence	Yes	34	16	626	67	33	15	507	63
	No	119	57	134	14	142	65	119	15
Vital status	Alive	171	82	721	77	193	88	642	80
	Deceased	38	18	207	22	25	11	157	20
Neoadjuvant treatment (rectal only)	Yes	-	-	-	-	159	84	437	68
	No	-	-	-	-	30	16	201	32
Adjuvant treatment *	Yes	108	52	362	39	124	57	342	43
	No	47	22	333	36	47	22	242	30
Behavioral Characteristics									
Smoking	Current	21	10	91	10	39	18	117	15
	Former	40	19	342	37	52	24	296	37
	Never	104	50	372	40	95	43	307	38
BMI, mean (SD)		29 (8)		29 (6)		28 (6)		28 (6)	
BMI categories	Underweight	2	1	15	2	8	4	12	2
	Normal weight	72	34	231	25	68	31	203	25
	Overweight	48	23	316	34	63	29	301	38
	Obese	62	30	327	35	63	29	233	29

* “Other” includes Asians, Native Hawaiians, Native Americans, and patients reporting belonging to more than one race. ^A^ Data has not yet been abstracted on *n* = 37 (2%) for ethnicity, *n* = 21 (1%) for race, *n* = 44 (0.2%) for tumor stage, *n* = 478 (22%) for recurrence, *n* = 8 (0.3%) for vital status, *n* = 191 (19%) for neoadjuvant treatment, *n* = 585 (26%) for adjuvant treatment, *n* = 302 (14%) for smoking, and *n* = 50 (6%) for BMI.

**Table 3:** Proportion of early- and late-onset colon cancer patients receiving adjuvant treatment by stage at diagnosis.

	Early-Onset Rectal Cancer			Late-Onset Rectal Cancer		
Stage at Diagnosis	Total *n*	Received Neoadjuvant Treatment	% Received Neoadjuvant Treatment	Total *n*	Received Neoadjuvant Treatment	% Received Neoadjuvant Treatment
Stage I	13	1	1	118	5	0.4
Stage II	31	14	45	179	49	27
Stage III	60	54	90	233	195	85
Stage IV	49	39	80	154	112	73

**Table 4:** Proportion of early- and late-onset rectal cancer patients receiving neoadjuvant and adjuvant treatment by stage at diagnosis (i.e., before the receipt of any neoadjuvant treatment).

	Early-Onset Rectal Cancer			Late-Onset Rectal Cancer		
Stage at Diagnosis	Total *n*	Received Neoadjuvant Treatment	% Received Neoadjuvant Treatment	Total *n*	Received Neoadjuvant Treatment	% Received Neoadjuvant Treatment
Stage I	10	4	40	81	26	32
Stage II	39	32	82	153	109	71
Stage III	101	92	91	297	230	79
Stage IV	34	29	85	90	68	76
	Total *n*	Received adjuvant treatment	% Received adjuvant treatment	Total *n*	Received adjuvant treatment	% received adjuvant treatment
Stage I	9	2	22	77	16	21
Stage II	36	28	78	136	77	57
Stage III	94	69	73	273	187	68
Stage IV	27	19	70	82	59	72

**Table 5:** Logistic regression OR (95% confidence interval) comparing tumor, clinical, and behavioral characteristics between early- vs. late-onset colorectal cancer patients.

Exposure Variable		Age of Onset N		Model 1 ^a^	Model 2 ^b^	Model 3 ^c^
		Early	Late			
Neoadjuvant treatment (rectal cancer only)	No	134	721	1.00	1.00	1.00
	Yes	319	524	2.45 (1.60–3.75)	2.25 (1.41–3.57)	2.30 (1.43–3.70)
Adjuvant treatment	No	133	453	1.00	1.00	1.00
	Yes	232	704	2.00 (1.53–2.62)	1.69 (1.25–2.28)	2.00 (1.43–2.81)
Stage at diagnosis	0	7	61	0.75 (0.33–1.74)	--	1.00 (0.46–2.17)
	I	50	322	1.00	--	1.00
	II	93	436	1.36 (0.93–1.98)	--	1.44 (0.97–2.13)
	III	185	597	1.98 (1.40–2.78)	--	1.99 (1.39–2.87)
	IV	107	291	2.31 (1.59–3.37)	--	2.50 (1.69–3.72)
Smoking	Ever	159	846	1.00	1.00	1.00
	Never	207	679	1.60 (1.27–2.03)	1.56 (1.23–1.99)	1.55 (1.21–1.98)
BMI	Underweight	62	95	1.26 (0.61–2.62)	1.02 (0.46–2.24)	1.08 (0.49–2.41)
	Normal weight	142	343	1.00	1.00	1.00
	Overweight	116	617	0.58 (0.44–0.77)	0.60 (0.45–0.80)	0.56 (0.41–0.76)
	Obese	128	561	0.72 (0.55–0.94)	0.78 (0.59–1.03)	0.66 (0.48–0.90)

^a^ adjusted for sex and race; ^b^ adjusted for sex, race, tumor site and stage; ^c^ mutually adjusted for sex, race, tumor site and stage, BMI, smoking, and study site.

## Data Availability

The data presented in this study are available in this article (and Supplementary Materials).

## Supplementary Materials

The following are available online at https://www.mdpi.com/article/10.3390/cancers13153817/s1, Table S1: Logistic regression OR (95% confidence interval) comparing tumor, clinical, and behavioral characteristics between early- (≤50 years), intermediate (50–55 years), and late-onset (≥55 years) colorectal cancer patients.
