# Supplementary material for: Clinical Characteristics and Outcomes of Colorectal Cancer in the ColoCare Study: Differences by Age of Onset
Source: Cancers (Basel). 2021 Jul 29;13(15):3817. doi: 10.3390/cancers13153817 (PMC8345133; doi:10.3390/cancers13153817)
Supplement: Supplementary file 1 [file cancers-13-03817-s001.zip › cancers-1244728-supplementary.pdf]

Supplementary Data

Supplementary Table S1. Logistic regression OR (95% confidence interval) comparing tumor, clinical, and behavioral characteristics between early- (≤50 years), intermediate (50-55 years), and late-onset (≥55 years) colorectal cancer patients.

**Exposure variable**		**Age of onset**					
		**Early-Onset**		**Intermediate onset**		**Late-onset**	
		**N**	**OR (95%CI)**	**N**	**OR (95%CI)**	**N**	**OR (95%CI)**
**Neoadjuvant treatment (rectal cancer only)**	**No**	134	REF	98	REF	623	REF
	**Yes**	319	2.55 (1.65-3.94)	98	1.24 (081-1.90)	426	REF
**Adjuvant treatment**	**No**	94	REF	72	REF	505	REF
	**Yes**	232	2.17 (1.61-2.85)	130	1.54 (1.15-2.05)	574	REF
**Stage at diagnosis**	**0**	7	0.91 (0.38-2.18)	10	1.30 (0.60-2.82)	51	REF
	I	50	REF	43	REF	279	REF
	**II**	93	1.44 (0.97-2.15)	55	0.99 (0.65-1.57)	381	REF
	**III**	185	2.06 (1.42-2.99)	98	1.22 (0.82-1.81)	499	REF
	**IV**	107	2.79 (1.86-4.18)	60	1.79 (1.16-2.78)	231	REF
**Smoking**	**Ever**	159	REF	110	REF	736	REF
	**Never**	207	1.74 (1.37-2.22))	130	1.54 (1.19-2.01)	549	REF
**BMI**	**Underweight**	62	0.98 (0.47-2.03)	16	--	79	REF
	**Normal weight**	142	REF	76	REF	358	REF
	**Overweight**	116	0.54 (0.40-0.71)	84	0.66 (0.48-0.90)	533	REF
	**Obese**	128	0.69 (0.52-0.91)	95	0.83 (0.61-1.14)	466	REF

mutually adjusted for sex, race.
